# Effects of chronic restraint stress in the prostate of prepubertal and adult rats

**DOI:** 10.1590/acb387123

**Published:** 2023-12-04

**Authors:** Isabella Mendes Procópio, Carina Teixeira Ribeiro, Roger Gaspar Marchon, Waldemar Silva Costa, Gabriela Faria Buys-Gonçalves, Francisco José Barcellos Sampaio, Marco Aurélio Pereira-Sampaio, Diogo Benchimol de Souza

**Affiliations:** 1 Universidade Estadual do Rio de Janeiro – Urogenital Research Unit – Rio de Janeiro (Rio de Janeiro) – Brazil.; 2 Universidade Federal Fluminense – Department of Morphology – Niteroi (Rio de Janeiro) – Brazil.

**Keywords:** Prostate, Rats

## Abstract

**Purpose:**

To investigate the effects of chronic stress in the prostate of prepubertal and adult rats.

**Methods:**

Thirty-two male rats were assigned into four groups depending on the type of treatment (control or stressed) and the age at which stress was initiated (prepubertal or adult). Restraint stress stimuli were applied for six weeks. Stressed prepubertal and adult rats evaluated immediately after the last stress stimuli were named SP and SA groups, respectively. Age-matched rats were used as control groups (CP and CA). At the end of the experiment, the rats were euthanized, and prostate morphological parameters were evaluated and statistically compared.

**Results:**

Application of stress stimuli to the SP group resulted in reduced body weight, but no prostate morphological modification was noted. The SA group showed reduced testosterone level and prostatic epithelium surface density, in comparison to CA group. Further, the prostatic lumen surface density was increased in adult stressed animals, in comparison to adult controls.

**Conclusions:**

The stress stimuli promoted changes in hormonal and morphological parameters in the prostate of adult stressed rats. Prepubertal stressed animals did not presented modifications of prostate morphology.

## Introduction

Stress is a biological condition to respond to an external stimulus. Although this condition is important enabling a better response to stressful situations, it involves several behavioral and physiological alterations^1^. Further when stress stimuli become persistent, a destructive effect on tissues are observed, and a set of morphological modifications are observed^1^^–^ 3.

One very known physiological response associated with stress conditions is the over-activation of the hypothalamus-hypophysis-adrenal axis, resulting in an increased secretion of glucocorticoids. Also, the hypothalamus-hypophysis-gonadal axis is influenced, with reduced sexual hormones secretion in chronic stress situations^4^.

Several studies have shown that the urogenital system organs have its morphology seriously altered by increased glucocorticoids levels and chronic stress^1^^,^ 5 ^–^ 9. However, few studies examinate the effects of stress on the prostate. Among then, some important experimental findings point to a relationship of stress stimuli and prostate cancer, with increased expression of cancer-related genes^10^, and accelerated prostate cancer development^11^^,^ 12.

The prostate morphology of stressed rats was superficially studied, with an apparent epithelium proliferation being observed in the ventral lobe^13^. But the prostatic stroma has not been studied, and the epithelium was not objectively assessed with morphometrical methods in stressed rodents. Further, it is already known that chronic stress can promote different alterations in the urogenital organs if induced during prepubertal or adult ages^5^^,^ 6, but this has not been investigated in the prostate yet.

Thus, the objective of the present study was to evaluate the prostate alterations in prepubertal and adult Wistar rats submitted to chronic stress.

## Methods

### Animals

Thirty-two male Wistar rats were used in the experiments. The rats were kept in a temperature-controlled room (22 ± 1°C) with an artificial dark–light cycle (lights on from 7 a.m. to 7 p.m.), and fed standard rat chow and water ad libitum. The Animal Care and Use Committee of the Universidade Estadual do Rio de Janeiro approved the handling of the animals and the study design (protocol number CEUA/004/2015), which were used in accordance with national and international regulations. The authors complied with the ARRIVE guidelines.

### Experimental design

Animals were assigned into four groups accordingly to age and treatment. SP (n = 7) was a group of 4-week-old prepubertal rats, and SA (n = 9) was a group of 10-week-old adult rats; both submitted to the stress stimuli. We compared each of these groups with age-matched control groups. CP (n = 8) was a control group of prepubertal rats, and CA (n = 8) was a control group of adult rats.

The SP and SA groups were submitted to chronic stress by the immobilization method^9^. Each animal was kept in a rigid opaque plastic tube for 2 hours daily, in the morning (from 9 to 11 a.m.), to restrain its movements during the six-week period. The plastic restraint tubes with different diameters and lengths were adjusted weekly depending on the rats’ size. The behavior of the animals during, and immediately after the stress period were observed and recorded. Meanwhile, the control groups (CP and CA) were kept under normal conditions and not submitted to any stresses, but food and water were removed during the same period (2 hours in the morning). All animals were euthanized 24 hours after the last day of stress stimuli application, when the rats were 10 (SP and CP) and 16 (SA and CA) weeks old. Euthanasia was performed by isoflurane (Isofluorano, BioChimico, Itatiaia, RJ, Brazil) inhalation in an induction chamber.

### Data collection

Just before death, body weight was measured, and blood was collected by heart puncture for the determination of testosterone levels using a commercially available enzyme-linked immunosorbent assay (ELISA) kit (Cat. ADI-900-065, Enzo, New York, United States of America, sensitivity of 5.67 pg/mL)^14^.

The ventral lobe of the prostate was dissected and fixed by immersion in formaldehyde 3.7% for at least 48 hours. Further, the samples were processed for paraffin embedding to obtain non-serial 5-?m-thick histological sections. Morphometric analyses were performed in hematoxylin and eosin (H&E) stained sections by a microscope (BX51, Olympus, Tokyo, Japan) coupled to a digital camera (DP70, Olympus). All images were saved in the tagged image file format (.tiff), at a resolution of 2,040 × 1,536 pixels^15^.

The height of the acinar epithelium was measured in micrometers with the “straight line” tool of the ImageJ software (National Institutes of Health, Bethesda, Maryland, United States of America) in photomicrographs of 200× magnification. For this purpose, 125 measurements of the epithelium (most commonly, five measurements per histological field, from at least 25 histological fields) were performed for each rat^16^^,^ 17.

The acinar area was measured in squared micrometers with the “polygon selection” tool of the ImageJ software in photomicrographs of 400× magnification. For this analysis, all acini that were completely observed in the histological field were measured^18^. At least, 25 histological fields per animal were studied.

The surface densities (Sv) of the lumen, epithelium and stroma were assessed by the point intercepts method^19^^,^ 20. Briefly, using the ImageJ software, a 100-point grid was superimposed over images under 400× magnification. Each structure touched by a point was counted, and its density was determined as a percentage of the analyzed field. For each rat, 25 fields were evaluated.

### Statistical analysis

The means of the groups submitted to the stress stimuli were compared with its correspondent age-matched control group using the Student’s t-test. All analyses were performed with the GraphPad Prism 5.0 software (GraphPad Software, San Diego, California, United States of America). Mean differences were considered significant when *p* < 0.05. All results were presented as mean ± standard deviation.

## Results

All animals submitted to stress stimuli showed behavioral alterations during and immediately after the stress sessions. The behaviors include attempts to scape before entering the tube, tachypnea, and diarrhea inside the tube, and running after being removed from the tube to the boxes.

### Effects of the stress stimuli in prepubertal rats

Body weight was reduced by 29 grams (14.1%; *p* < 0.05) in SP animals in comparison to their controls. No significant difference of serum testosterone levels was identified between SP and CP groups (*p* = 0.23). Also, no morphometrical differences were observed among these groups regarding epithelium height (*p* = 0.55), acinar area (*p* = 0.27), lumen Sv (*p* = 0.89), epithelium Sv (*p* = 0.61), and stroma Sv (*p* = 0.49). All numerical data of groups CP and SP are presented in Table 1.

**Table 1:** Quantitative data of prepubertal rats submitted to chronic stress (SP) and its age matched control group (CP)*.

Parameters	CP	SP	*P*-value
Body weight (g)	206.23 ± 11.02	177.13 ± 15.85	< 0.01
Testosterone serum level (ng/mL)	13.00 ± 1.67	11.93 ± 1.21	0.23
Prostatic epithelium height (μm)	21.27 ± 2.39	20.57 ± 1.98	0.55
Prostatic acinar area (×10^3^ μm^2^)	50.88 ± 10.96	59.23 ± 11.32	0.27
Prostatic lumen surface density (%)	48.64 ± 9.24	48.04 ± 8.07	0.89
Prostatic epithelium surface density (%)	45.30 ± 5.85	43.51 ± 7.36	0.61
Prostatic stroma surface density (%)	6.84 ± 1.08	7.23 ± 1.07	0.49

* Data expressed as mean ± standard deviation. Source: Elaborated by the authors.

### Effects of the stress stimuli in adult rats

No difference in body weight was observed when comparing CA with SA (*p* = 0.86). The testosterone serum levels of stressed animals showed reduction of 2.42 ng/mL, what represents 21.5% of this hormonal level (*p* = 0.04).

**Figure 1:**
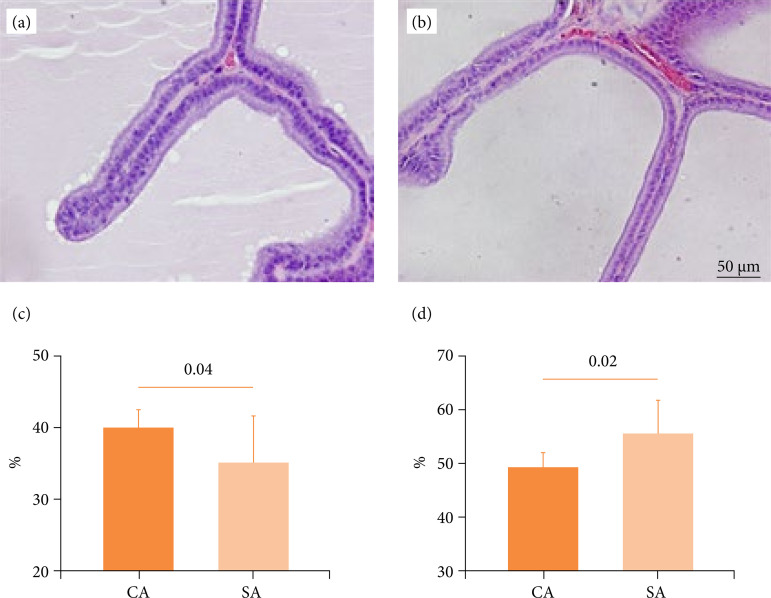
Illustrative images from control and stressed rats. **(a)** Photomicrography of prostate from adult control rat (group CA); hematoxylin and eosin, 400×. **(b)** Photomicrography of prostate from adult stressed rat (group SA); hematoxylin and eosin, 400×. **(c)** Graphic showing the reduction of prostatic epithelium surface density in group SA in comparison to group CA. **(d)** Graphic showing the augmented prostatic lumen surface density in group SA in comparison to group CA.

Source: Elaborated by the authors.

In respect to epithelium height (*p* = 0.58), acinar area (*p* = 0.10), and stroma Sv (*p* = 0.61), no statistical difference was observed among groups CA and SA. Meanwhile, stressed animals showed an increase of 12.0% in lumen Sv (*p* = 0.02), in comparison to its age-matched control group. Further, the prostatic epithelium Sv of group SA was reduced by 12.6% (*p* = 0.04) in comparison to CA (Fig. 1). All numerical data of groups CP and SP are presented in Table 2.

**Table 2:** Quantitative data of adult rats submitted to chronic stress (SA) and its age matched control group (CA)*.

	CA	SA	*p*-value
Body weight (g)	353.21 ± 32.80	342.37 ± 28.39	0.86
Testosterone serum level (ng/mL)	11.25 ± 2.14	8.83 ± 1.53	0.04
Prostatic epithelium height (?m)	19.20 ± 2.15	19.82 ± 2.25	0.58
Prostatic acinar area (×10^3^ ?m^2^)	62.36 ± 5.73	74.52 ± 19.14	0.10
Prostatic lumen surface density (%)	49.65 ± 2.12	55.62 ± 6.39	0.02
Prostatic epithelium surface density (%)	40.30 ± 2.14	35.22 ± 6.18	0.04
Prostatic stroma Sv (%)	8.67 ± 0.49	8.07 ± 1.74	0.35

* Data expressed as mean ± standard deviation. Source: Elaborated by the authors.

## Discussion

The present study shows that adult rats submitted to chronic stress shows prostatic and hormonal alterations, with testosterone reduction, prostatic acinar area and lumen surface density augmentation and prostatic epithelium reduction. Meanwhile, no statistical difference (in testosterone levels and prostate morphology) was observed in prepubertal rats submitted to chronic stress.

Chronic stress is known to activate the hypothalamic-pituitary-adrenal axis, with consequences on the hypothalamic-pituitary-gonadal axis. One main result of this endocrine disturbance is the reduction of sexual hormones production/secretion in stressed individuals^21^. Our group have already shown that this model of chronic stress reduces the testosterone serum levels in chronically stressed rats^5^^,^ 6 ^,^ 9, similarly of what was observed in the current experiment. This is in accordance with what Selye proposed when studying the mechanisms of stress. During stressful situations, the whole organism prepares to deal with the stressor, and physiological mechanisms not required in that immediately time are suppressed^22^. The reproductive system is one example of a physiological system that should theoretically be suppressed in a stressful situation^23^. Glucocorticoids can also interfere with testosterone levels directly, and it is known that the hypothalamic–pituitary–adrenal and the hypothalamic–pituitary–gonadal axis interact with each other^21^.

The testosterone reduction led to penile and testicular morphological modifications^5^^,^ 6 ^,^ 9, but the prostate gland was not studied yet. As an androgen-dependent organ, the prostate is very influenceable by sexual hormones, and it was expected that the prostate gland would be very modified by chronic stress. Surprisingly, it was observed only discrete morphological modifications in our stressed animals’ prostate. More prolonged periods of stress would be interesting to verify if more pronounced modifications could be observed in adult, and (specially) in prepubertal animals. This study used the same protocol (2 hours per day, for six weeks) used before with interesting results in other urogenital organs^1^^,^ 5 ^–^ 7 ^,^ 9. Thus, it is possible to believe that the prostate may be a less stress-affected organ.

In adult rats submitted to chronic stress, a reduction of prostatic epithelium surface density was observed. This can be a direct effect of stress on the epithelial cells or (more probably) an indirect effect, i.e., a consequence of the reduced testosterone level. Paradoxically, the epithelium height was not modified in stressed rats. This could be explained by the reduced number of epithelial folds in stressed animals, leading to a reduced surface occupied by the epithelium, without epithelial height modification. Another finding in stressed animals was the raised luminal surface density, which was probably a consequence of the epithelium Sv reduction.

The study of Mukerjee and Rajan^24^ showed very comparable findings with those from the present study. In their study, prepubertal rats stressed by maternal deprivation or foot-shock showed reduced epithelium volume in comparison to control animals. In the study of Morone Pinto et al.^25^, chronic stress (induced pharmacologically) also negatively impacted the prostatic epithelium, reducing its height. Although these authors did not measure the Sv of epithelium, these findings corroborate the outcomes that chronic stress negatively impacts the prostate epithelium.

One interesting result of the current study is that adult animals showed more prominent morphological alterations in the prostate when compared to prepubertal rats. Actually, no statistically significant difference was observed in the prostate of prepubertally stressed rats, in comparison to controls. One possible reason would be the fact that prepubertal individuals are more adaptable to different situations than adults. Pubertal phase is mainly a phase of important changes and adaptation. Further, prepubertal animals may not suffer alterations of the hypothalamic-pituitary-gonadal axis as testicles are not fully developed. This could explain the non-statistically reduced testosterone levels in group SP. These findings are similar to our previous studies which compared the stress in prepubertal and adult rats^5^^,^ 6. When studying the testes and the penis, we observed that the damages promoted by chronic stress are more significant when stress stimuli is induced in adulthood than during prepubertal phase. The only alteration observed in prepubertal animals submitted to chronic stress was a body weight reduction. This could be explained by an accentuated energy expenditure caused by stress, although future studies should be performed to better elucidate this finding.

In the present study, rats were used to evaluate the effects of chronic stress on the prostate. A limitation of this study is the animal model used, as the results may not reflect the actual effects in men. Even considering that the prostatic ventral lobe of rats is a good model for morphological studies, several differences are observed among the prostate of human and rats.

Further studies on the effects of chronic stress are warranted. Different types, intensity, and time of the stressor stimulus, as well as the age of animals, would be necessary to be studied before a definitive decision regarding the impact of stress on the prostate. Although corticosterone analysis was not performed in this study, which may be considered a limitation of the study, the stress induction method is a well stablished protocol. Our findings showed that chronic stress applied during adulthood causes prostatic morphological alterations. However, it is not known if these alterations could be reversed upon withdrawal of the stressor stimulus or prevented by some therapy. One possible limitation regarding all stress models is the adaptation to the stress stimuli. As behavior alterations is noted in all animals of stressed groups, throughout the experiment, it is possible to believe that this was not the case in the present study.

## Conclusion

Based on the results obtained in the present study, it was possible to state that the stress stimuli promote hormonal disbalances and morphological alterations in the prostate of adult rats. Meanwhile, animals stressed during prepubertal phase did not presented modifications of prostate morphology or testosterone secretion alterations.

## References

[1] Benchimol de Souza D, Silva D, Silva CMC, Sampaio FJB, Costa WS, Cortez CM (2011). Effects of immobilization stress on kidneys of Wistar male rats: a morphometrical and stereological analysis. Kidney Blood Press Res.

[2] Rosmond R, Bjorntorp P (2000). The hypothalamic-pituitary-adrenal axis activity as a predictor of cardiovascular disease, type 2 diabetes and stroke. J Intern Med.

[3] Ennab W, Mustafa S, Wei Q, Lv Z, Kavita NMX, Ullah S, Shi F. (2019). Resveratrol Protects against Restraint Stress Effects on Stomach and Spleen in Adult Male Mice. Animals.

[4] Rivier C, Rivest S. (1991). Effect of stress on the activity of the hypothalamic-pituitary-gonadal axis: peripheral and central mechanisms. Biol. Reprod.

[5] Ribeiro CT, Costa WS, Sampaio FJB, Pereira Sampaio, De Souza DB (2019). Evaluation of the effects of chronic stress applied from the prepubertal to the adult stages or only during adulthood on penile morphology in rats. Stress.

[6] Ribeiro CT, De Souza DB, Costa WS, Sampaio FJ, Pereira-Sampaio MA (2018). Immediate and late effects of chronic stress in the testes of prepubertal and adult rats. Asian J Androl.

[7] Marchon R, Ribeiro CT, Costa WS, Sampaio FJB, Pereira-Sampaio MA, Souza DB (2018). Immediate and Late Effects of Stress on Kidneys of Prepubertal and Adult Rats. Kidney Blood Press Res.

[8] Ribeiro GS, De Souza DB, Cortez CM, Silva D, Costa WS, Sampaio FJB (2014). Effects of prepubertal corticosterone treatment on urinary bladder. Acta Cir Bras.

[9] De Souza DB, Silva D, Cortez CM, Costa WS, Sampaio FJB (2012). Effects of Chronic Stress on Penile Corpus Cavernosum of Rats. J Androl.

[10] Flores IE, Sierra-Fonseca JA, Davalos O, Saenz LA, Castellanos MM, Zavala JK, Gosselink KL (2017). Stress alters the expression of cancer-related genes in the prostate. Stress alters the expression of cancer-related genes in the prostate.

[11] Hassan S, Karpova Y, Baiz D, Yancey D, Pullikuth A, Flores A, Register T, Cline JM, D’Agostino R, Danial N, Datta SR, Kulik G. (2013). Behavioral stress accelerates prostate cancer development in mice. J Clin Invest.

[12] Bellinger DL, Dulcich MS, Molinaro C, Gifford P, Lorton DS, Gridley DS, Hartman RE (2021). Psychosocial Stress and Age Influence Depression and Anxiety-Related Behavior, Drive Tumor Inflammatory Cytokines and Accelerate Prostate Cancer Growth in Mice. Front Oncol.

[13] Huang S, Fang X, Meng Y, Chen Y, Zhang X, Zhao S. (2009). Sympathetic nervous system overactivity in the Wistar rat with proliferative lesions of ventral prostate induced by chronic stress. Urol Int.

[14] Ribeiro CT, Milhomem R, De Souza DB, Costa WS, Sampaio FJB, Pereira-Sampaio MA (2014). Effect of antioxidants on outcome of testicular torsion in rats of different ages. J Urol.

[15] Ribeiro CT, De Souza DB, Costa WS, Pereira-Sampaio MA, Sampaio FJ (2015). Effects of testicular transfixation on seminiferous tubule morphology and sperm parameters of prepubertal, pubertal, and adult rats. Theriogenology.

[16] Felix-Patrício B, Miranda AF, Medeiros JL, Gallo CBM, Gregório BM, Souza DB, Costa WS, Sampaio FJB (2017). The prostate after castration and hormone replacement in a rat model: structural and ultrastructural analysis. Int Braz J Urol.

[17] Pinto FC, Costa WS, Silva PC, de Souza DB, Gregório B, Sampaio FJB (2016). Effects of L-Glutamine oral supplementation on prostate of irradiated rats. Int Braz J Urol.

[18] Pinto FC, Campos-Silva P, Souza DB, Costa WS, Sampaio FJ (2016). Nutritional supplementation with arginine protects radiation-induced effects. An experimental study. Acta Cir Bras.

[19] Vargas RA, Oliveira LP, Frankenfeld S, Souza DB, Costa WS, Favorito LA, Sampaio FJB (2013). The prostate after administration of anabolic androgenic steroids: a morphometrical study in rats. Int Braz J Urol.

[20] Felix-Patrício B, De Souza DB, Gregório BM, Costa WS, Sampaio FJ (2015). How to Quantify Penile Corpus Cavernosum Structures with Histomorphometry: Comparison of Two Methods. Biomed Res Int.

[21] Toufexis D, Rivarola MA, Lara H, Viau V. (2014). Stress and the reproductive axis. J Neuroendocrinol.

[22] Chichinadze K, Chichinadze N. (2008). Stress-induced increase of testosterone: contributions of social status and sympathetic reactivity. Physiol Behav.

[23] Bedgood D, Boggiano MM, Turan B. (2014). Testosterone and social evaluative stress: the moderating role of basal cortisol. Psychoneuroendocrinology.

[24] Mukerjee B, Rajan T. (2004). Morphometric study of rat prostate in normal and under stressed condition. J Anat Soc India.

[25] Morone Pinto, Silva D, Silva CS, Silva PC, De Souza DB, Sampaio FJB, Costa WS, Cortez CM (2016). Prepubertal Exposure to Pharmacological Stress Induces Structural Alterations in the Prostate of Rats. JSM Biol.

